# Manual Acupuncture for Treatment of Diabetic Peripheral Neuropathy: A Systematic Review of Randomized Controlled Trials

**DOI:** 10.1371/journal.pone.0073764

**Published:** 2013-09-12

**Authors:** Wei Chen, Guo-yan Yang, Bo Liu, Eric Manheimer, Jian-Ping Liu

**Affiliations:** 1 Centre For Evidence-Based Chinese Medicine, Beijing University of Chinese Medicine, Beijing, China; 2 Medical Care Center, Beijing Friendship Hospital, Capital Medical University, Beijing, China; 3 Center for Integrative Medicine, University of Maryland School of Medicine, Baltimore, Maryland, United States; Iran University of Medical Sciences, Iran (islamic Republic Of)

## Abstract

**Objective:**

Manual acupuncture has commonly been used in China, either alone or in combination with conventional medicine, to treat diabetic peripheral neuropathy (DPN). The objective of this study was to perform a systematic review to evaluate the potential benefits and harms of manual acupuncture for DPN to justify its clinical use.

**Methods:**

We searched for published and unpublished randomized controlled trials of manual acupuncture for DPN till 31 March 2013. Revman 5.2 software was used for data analysis with effect estimate presented as relative risk (RR) and mean difference (MD) with a 95% confidence interval (CI).

**Results:**

A total of 25 trials involving 1649 participants were included. The methodological quality of included trials was generally poor. Meta-analysis showed that manual acupuncture had better effect on global symptom improvement compared with mecobalamin (RR 1.31, 95%CI 1.21 to 1.42), vitamin B1 and B12 (RR 1.55, 95%CI 1.33 to 1.80), and no treatment (RR 1.56, 95%CI 1.31 to 1.85), and that the combination of manual acupuncture and mecobalamin had better effect compared with mecobalamin alone on global symptom improvement (RR 1.56, 95%CI 1.28 to 1.90). Adverse events were not reported in any trials. The asymmetric funnel plot suggested publication bias.

**Conclusions:**

Despite the number of trials of manual acupuncture for DPN and their uniformly positive results, no clinically relevant conclusions can be drawn from this review due to the trials’ high risks of bias and the possibility of publication bias. Clearly defined and internationally acknowledged outcome measures are required for future study. There remains an urgent need for training Chinese researchers in conducting unbiased trials as well as prospectively registering all initiated Chinese trials to avoid publication bias.

## Introduction

World Health Organization (WHO) data suggest that there will be 353 million people with diabetes mellitus by 2030 [1]. Diabetic peripheral neuropathy (DPN) is one of the most common complications of diabetes mellitus. Population based cohort studies have shown that 66% of people with type 1 diabetes and 59% of people with type 2 diabetes have objective evidence of peripheral neuropathy [2]. DPN is a chronic progressive disease, characterized by a progressive loss of nerve fibres that predisposes the person to painful or insensitive extremities, neuropathic ulceration and amputation, and results in a large disease burden in terms of incapacity for work, poor quality of life and consumption of health care resources.

Acupuncture is an alternative medicine therapy originating in ancient China, and it has been used since ancient times to treat symptoms of diabetic neuropathy. Acupuncture hinges on the belief that a person’s state of health depends on the balance and level of energy in the body, and that stimulating certain acupoints can correct imbalances in the flow of Qi through channels known as meridians and thereby restore balance to the body. There are different types of acupuncture in China, including manual body acupuncture, scalp acupuncture, auricular acupuncture, electro-acupuncture, acupoint injection, moxibustion (an external method of preventing and treating diseases by ignition of moxa to stimulate the acupuncture points), and so on. Among them, manual acupuncture is more tradition and widely used in practice. Many Traditional Chinese Medicine (TCM) practitioners consider this practice is closer to TCM theory because it allows individualized treatment for different syndrome of TCM diagnosis, and is recommended for the treatment of DPN [3]. Manual acupuncture refers to treating patients by inserting thin, solid needles into acupuncture points (acupoint) on the skin. The needles are often manipulated by the practitioner, with the intention of eliciting the Deqi sensation (i.e., a pain, achiness, stinging, or dullness at the needle insertion site, which is an indicator that the acupuncture needle has been correctly placed).

Although many trials of manual acupuncture for DPN have been published, these trials have not yet been systematically reviewed. Therefore, we conducted a systematic review of randomized trials to assess the benefit and harm of manual acupuncture on DPN.

## Methods

### Protocol and Registration

A protocol of this systematic review was published in the PROSPERO database (identification number: CRD42013004191) [4].

### Search Strategy and Study Selection

Literature searches were conducted in the CENTRAL of the Cochrane Library (2012, Issue 12), MEDLINE, EMBASE, SinoMed, Chinese National Knowledge Infrastructure (CNKI), Chinese VIP information (VIP), China’s Important Conference Papers Database, and China’s Dissertation Database from their inception to 31 March 2013. The following search terms were used individually or combined: ‘zhen ci’ (acupuncture), ‘tang niao bing shen jing bing bian’ (diabetic neuropathy), ‘tang niao bing zhou wei shen jing bing bian’ (diabetic peripheral neuropathy)’, ‘acupuncture’, ‘acupuncture therapy’, ‘needling’, ‘diabetic peripheral neuropathy’, ‘diabetic neuropathy’, ‘diabetic peripheral neuropathy’, ‘DPN’, ‘clinical trial’, and ‘randomized controlled trial’.

Two authors conducted the literature searching (WC, GYY), study selection (WC, BL), and data extraction (WC, BL) independently. The extracted data included authors and title of study, year of publication, study size, age and gender of the participants, details of methodological information, details of needling, treatment regimen, details of the control interventions, outcomes, and adverse effects for each study. Disagreement was resolved by discussion and consensus reached through a third party (JPL).

### Inclusion Criteria

We included parallel randomized controlled trials (RCTs) that evaluated manual acupuncture for the treatment of DPN, regardless of language or publication status.

The definition of DPN must conform to the following diagnostic criteria: the patient has diabetes mellitus by internationally recognized criteria, such as the WHO criteria [5]; and the patient has a predominantly distal symmetrical sensorimotor polyneuropathy of the limbs; other causes of sensorimotor polyneuropathy have been excluded.

We defined manual acupuncture as manual stimulation of acupuncture points, with penetration of the skin by thin metal needles. Scalp acupuncture, acupoint injection, electroacupuncture, laser acupuncture, moxibustion, or the combination of manual acupuncture and the above were excluded. We included trials only if the treatment was given for a minimum of four weeks.

Eligible control groups were another (potentially) active treatment, sham acupuncture, or no treatment at all. We also included RCTs that compared acupuncture plus another (potentially) active treatment versus that other (potentially) active treatment alone were also included.

The primary outcome was global symptom improvement measured by a validated instrument such as a visual analog (VAS) scale [6], or total symptom score [7]. Where this outcome was not available, we used the global symptom improvement measured by whatever criteria were used by the authors as the primary outcome. Secondary outcomes were change in nerve conduction velocity measured by validated methods, quality of life, and adverse events.

### Trial Quality Assessment

Two authors (WC, GYY) evaluated the quality of included trials. The quality of included trials were assessed by using the risk of bias tool according to the ‘Cochrane Handbook of Systematic Reviews of Interventions’ (Chapter 8) to address the following five criteria [8]: random sequence generation, allocation concealment, blinding of participants and personnel, blinding of outcome assessment, incomplete outcome data, and selective reporting.

### Reporting Quality Assessment

We evaluated the reporting quality of the included RCTs based on 25 items of CONSORT Statement, 2010 [9] and six components from STRICTA 2010 [10]. We responded with ‘yes’ or ‘no’ to each item to judge whether the authors had reported, or had recorded concrete details of the reports accomplished in accordance with the requirement of each item. The number of reports which met the standards of CONSORT 2010 and STRICTA 2010 was counted and the percentage of application of each item was calculated.

### Data Analysis

Data were summarized using relative risk (RR) with 95% confidence intervals (CI) for binary outcomes or mean difference (MD) with 95% CI for continuous outcomes. Revman 5.2 software was used for data analyses. Meta-analysis was performed if the trials had a good homogeneity on study design, participants, interventions, control, and outcome measures, which was assessed by examining I2 (a quantity that describes approximately the proportion of variation in point estimates due to heterogeneity rather than sampling error). Fixed-effect model was used for meta-analysis. If at least ten trials were available for a meta-analysis, we assessed for the likelihood of publication bias by constructing funnel plots [8]. If we identified a sufficient number of randomized trials, we had planned to perform sensitivity analyses to explore the influence of trial quality on effect estimates. The quality components of methodology included adequacy of generation of allocation sequence, concealment of allocation, double blinding, and the use of intention-to-treat (yes or no).

## Results

### Description of Studies

**Figure 1:**
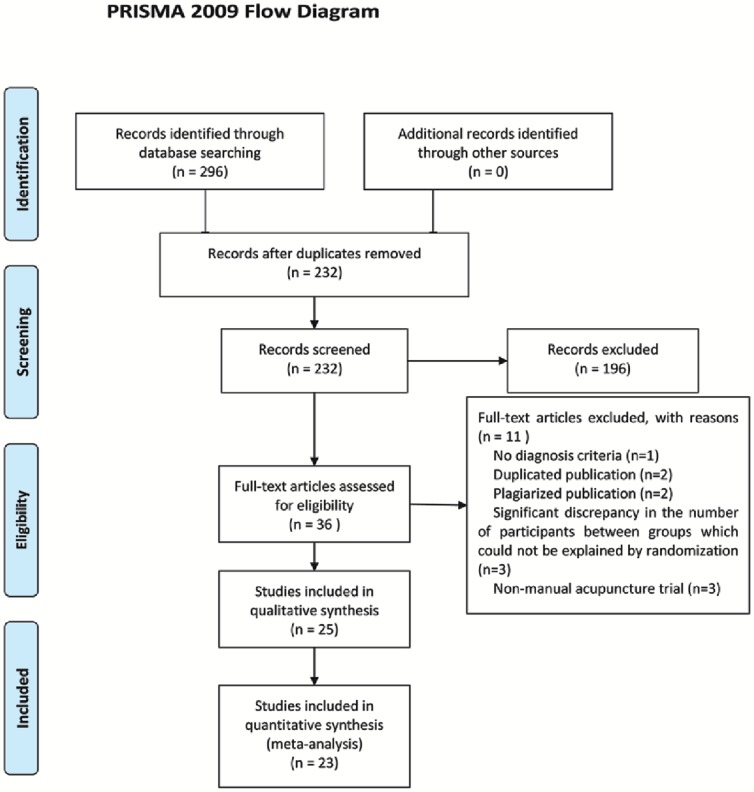
PRISMA 2009 Flow Diagram.

A flow chart depicted the search process and study selection (Fig 1). After primary searches from the ten databases, 296 citations were screened. After reading the titles and abstracts, a majority of them was excluded. Full texts of 36 papers were retrieved, and finally 25 RCTs were included [11]–[35] including two three-armed RCTs [28], [34]. However, because the intervention other than manual acupuncture in the two three-armed RCTs is moxibustion [34] and tapping collaterals with skin needles [28], only data of manual acupuncture was collected and analyzed.

The characteristics of included trials were listed in Table S1. All trials were conducted in China, and published in Chinese. A total of 1649 participants with DPN were involved, with an average number of 66 per trial, ranging from 28 to 100. No trial reported sample size calculation. Four trials included DPN patients of both type 1 and 2 DM [15]–[17], [23], six trials included DPN patients of type 2 DM [18], [22], [24]–[26], [29] and the remaining 15 trials did not mention the type of DM they included. For the diagnostic criteria of diabetes, 18 trials used the WHO diagnostic criteria [11]–[16], [18]–[20], [22], [24]–[27], [29]–[31], [33], six trials used the diagnostic criteria of the American Diabetes Association (ADA) [17], [23], [28], [32], [34], [35], and one trial used the criteria of the China Guideline for Diabetes Prevention and Treatment [21]. Six trials provided information on patients’ syndrome differentiation (Bianzheng, TCM diagnosis) [19], [20], [22], [24], [32], [33]. The details of manual acupuncture were shown in Table S2. The control interventions included B vitamins, namely, mecobalamin (11 trials) [11], [13], [14], [17], [19]–[21], [32]–[35], vitamin B1 and B12 (five trials) [15], [22]–[25], inositol (one trial) [31], and no treatment (four trials) [17], [26], [28], [30]. Four trials compared the combination of manual acupuncture and mecobalamin with mecobalamin alone [16], [18], [27], [29]. No included trial used sham acupuncture as the control. Hypoglycemic therapy was used as co-intervention in all the included trials, including oral hypoglycemic drug, insulin, exercise, etc.

The outcomes reported included global symptom improvement (23 trials), and change in motor/sensory nerve conduction velocity (15 trials). For the outcome of global symptom improvement, because the dichotomous outcomes of global improvement of included RCTs were presented in the form of multiple strata and different cut point were used, therefore we combined all positive outcomes into a single positive category (i.e., improvement) and the remaining strata constituted the negative category (i.e., no improvement). For the outcome of nerve conduction velocity, the nerves that were measured were diverse, as shown in Table S3. No trial reported incidence of complications, quality of life, health economics, adverse events, or follow-up after the end of intervention.

### Methodological Quality

The majority of the included trials were assessed to be of general poor methodological quality according to the predefined quality assessment criteria. Although ‘random allocation’ was mentioned in all trials, only 7 trials described the methods for random sequence generation including random number table [13], [14], [21], [23], [29], [34] and computer software [28]. In addition, insufficient information was provided to judge whether or not it was conducted properly. Allocation concealment was not reported in any RCT. Blinding was also not reported in any RCT. No trial reported drop-outs or mentioned intention-to-treat analysis. Selective reporting was generally unclear in the RCTs due to the inaccessibility to the trial protocol.

### Reporting Quality Assessment

The reporting quality assessment of 25 RCTs according to CONSORT 2010 and STRICTA 2010 were shown in Table S4 and Table S5, respectively. While all 25 RCTs reported information on the CONSORT checklist items related to interventions and outcomes (Table S4), few RCTs adequately reported the study methodology-related items such as randomization, blinding, and methods of handling losses to follow-up. In addition, despite the fact that the general descriptions of the ‘interventions’ met the CONSORT criteria for all trials, some more detailed STRICTA intervention-related items (e.g., practitioner background, any co-interventions) still remained unreported for all trials.

### Effect Estimates

The effect estimates of manual acupuncture were shown in Table S3.

#### 1. Effect on global symptom improvement

Twenty-three trials reported the effect of manual acupuncture on global symptom improvement [11]–[25], [28]–[35]. Meta-analysis showed better effect of manual acupuncture compared with mecobalamin [11], [13], [14], [17], [19]–[21], [32]–[35], vitamin B1 and B12 [15], [22]–[25], and no treatment [12], [28], [30]. Meta-analysis showed better effect of the combination of manual acupuncture and mecobalamin compared with mecobalamin alone [16], [18], [29]. One trial [31] showed better effect of manual acupuncture compared with inositol.

The forest plot of comparisons of manual acupuncture versus conventional medicine and manual acupuncture plus cobamamide versus cobamamide alone for the outcome of global symptom improvement were shown in Figures S1 and S2, respectively.

#### 2. Effect on nerve conduction velocity

Fourteen trials reported the effect of manual acupuncture on nerve conduction velocity [11]–[13], [16], [17], [19], [22], [23], [25]–[27], [29], [32], [33], [35]. However, the nerves that were measured were quite diverse, a total of eight different nerves were measured. All demonstrated the effect favoring manual acupuncture except one [12].

### Adverse Events

No trial reported adverse events.

### Funnel Plot Analysis

The funnel plot demonstrated asymmetry, suggesting publication bias (Figure S3).

### Sensitivity Analysis

Sensitivity analyses were not conducted due to the fact that all the included RCTs were of high risk of bias. Although 7 RCTs stated methods for random sequence generation, insufficient information was provided to judge whether or not it was conducted properly. Allocation concealment, blinding, and the use of intention-to-treat were not mentioned in any RCT.

## Discussion

This review suggests that manual acupuncture may have beneficial effects for the treatment of DPN. However, these positive findings should be interpreted cautiously due to the high risks of bias of all included trials, the possibility of publication bias, and the variability of acupuncture protocols.

The poor methodology of included RCTs prevents drawing conclusions that justify the clinical use of manual acupuncture for DPN. In this review, all the trials were evaluated as ‘high risk of bias’, which has also been found in previous studies [36], [37]. In addition, the poor methodology of included trials prohibited us from performing meaningful sensitivity analysis as planned. Namely, we were unable to determine the robustness of the results of the review to the exclusion of the trials with inadequate methodology due to the fact that all the included trials were of ‘inadequate methodology’.

Only seven RCTs stated the randomization procedures that were used, while the remainder only mentioned that ‘the patients were randomized into two groups’ with no further information. However, these 7 RCTs provided insufficient information to judge whether the randomization was conducted properly. Based on methodological research evaluating the authenticity of ‘claimed’ randomized trials published in China [38], there was a strong possibility that some of these claimed RCTs were not real RCTs.

The type of control used may be the major source of bias, particularly for the global improvement outcome, which was subjective and patient-reported. All included RCTs compared acupuncture versus B vitamins or no treatment. No sham acupuncture was used. Participants in these trials are not blinded, and therefore know whether they were getting acupuncture or the vitamin control. In addition, participants in acupuncture trials might have enrolled because they have expectations for a benefit of acupuncture and would be unlikely to have strong *a priori* preferences or expectations of benefit from vitamins, which most DPN patients would likely have easy access to, even without participating in a trial. If participants have pre-treatment preferences for acupuncture relative to the vitamin control treatment, or expectations of greater improvements from acupuncture than vitamins, these preferences and expectations may have positively biased acupuncture participants' later responses to questions about their overall global improvement. Indeed, methodological research suggests that acupuncture may be associated with greater expectation effects than standard therapies [39]–[44]. However, one thing need to be mentioned is that while differences in expectations may explain much of the positive benefit for the global symptom outcome, the objective outcome of nerve conduction velocity, in contrast, is much less likely to be affected by participants’ expectations of a benefit of acupuncture [45]. That is, it is unlikely that the participants’ knowledge of whether they were receiving acupuncture or vitamins would affect their nerve conduction velocity. Therefore, the uniformly positive results for the nerve conduction velocity outcome were compelling, even in the absence of patient blinding.

Another possible source of bias might be incomplete outcome report. We believe that drop out or withdrawal is inevitable in the course of clinical research. However, although the treatment duration of these 25 trials ranged from 4 to 12 weeks, no trial reported drop-out or withdrawal, or mentioned intention-to-treat analysis. Possibility existed that some trials did not reported these missing data. If the missing data was not comparable between groups, this would lead the exaggerated treatment effect of manual acupuncture [46]. In addition, no trial reported follow-up after the treatment, therefore the long-term effect of acupuncture could not be established.

Our review found highly diversity in manual acupuncture in included RCTs which specified mainly on the acupoints selected and specific manipulation, as shown in Table S3. This is mainly because in China, acupuncturists believed that treatment should be given based on individualized (tailored) syndrome pattern. However, in this review, only six trials provided information on patients’ syndrome differentiation. Emphasis should be paid to encourage authors to explain each syndrome differentiation (‘Bianzheng’ in Chinese) by using common medical terms in the future trials, therefore making it understandable by physicians and consumers. The diversity of manual acupuncture should also be kept in mind when interpreting the results of meta-analysis.

One thing need to mention was that, besides the control of no treatment, all the other trials in this review compared acupuncture versus B vitamins, namely, vitamin B12, B1, mecobalamin, or inositol. The effectiveness of vitamin B12 on DPN has been showed by RCTs and systematic review [47], [48]. However, there was no sufficient evidence so far to support the efficacy of mecobalamin and inositol [49]–[51]. We should cautiously interpret these positive results of manual acupuncture when they were compared with mecobalamin or inositol.

For outcome, although almost all RCTs reported global symptom improvement, we found that different criteria or cut point were used in included RCTs. Most of the criteria were self defined and lack of unambiguous definition on cut point, which made it difficult to interpret the effects of acupuncture even if the result was positive. Clearly defined and internationally acknowledged outcome measures are required for future study. In this review we used one cut point, namely, to combine all positive outcomes into a single positive category (i.e., improvement) and the remaining into negative category (i.e., no improvement). That might be the possible reason if our result was different from those of original studies.

The report of adverse events of manual acupuncture was not adequate in the review, though we have to admit that a systematic review of RCTs is not the optimal design for identifying rare but serious harms [8]. No trial reported adverse events, which was coincident with previous studies. Turner found out that adverse events are often not well-reported in CAM RCTs [52]. Incidence rates for major adverse effects of acupuncture are best estimated from large prospective surveys of practitioners. Four such surveys of acupuncture safety have been conducted, two in Germany [53], [54] and two in the United Kingdom [55], [56], which confirmed that serious adverse events after acupuncture are uncommon. However, future investigator or acupuncturist should be encouraged to monitor and report adverse events in clinical trials to evaluate the potential harms of manual acupuncture.

The funnel plot demonstrated asymmetry, suggesting potential publication bias. All the included trials were conducted in China and published in Chinese. No papers in English were identified, and no ‘negative’ study was included. We undertook extensive searches for unpublished material, but found no unpublished ‘negative’ studies. The preferential publication of positive studies might be due to the lack of awareness to register clinical trials in China, the rejection of journal editors to negative trials, and the inaccessibility to unpublished data. Several initiatives are underway with the objective of insuring that all trials, both positive and negative, will be published in the future. These include efforts to promote the prospective registration of all clinical trials, publication of clinical trial protocols, and reporting of negative clinical trials [57]. In addition, the funnel plot also demonstrated that the asymmetrical plot also partly result from that some smaller studies were of lower methodological quality and therefore produce exaggerated intervention effect estimates.

In our review, we also assessed the quality of repots of included RCTs according to the CONSORT and STRICTA statement. We noticed that a study that assessed the quality of reports about RCTs of acupuncture treatment on DPN has been published in the year 2012 [58]. Our results differ from this study mainly on the assessment based on STRICTA. In Chen Bo’s study [58], the RCTs were lacking in details of needling, details of other intervention, practitioner background and so on; while in our review, the reports on details of needling were satisfactory. The reason for this difference between the two reviews might be explained by the different inclusion criteria of the two reviews. In Chen Bo’s study [58], they included all types of acupuncture, including manual acupuncture, acupoint injection, scalp acupuncture and etc. We only included manual acupuncture, and because our aim is to assess the efficacy and safety of manual acupuncture on DPN, we used more strict inclusion criteria than Chen Bo’s study; therefore, we included fewer RCTs than Chen Bo’s study. In general, papers of manual acupuncture are more likely to provide detailed information on acupoints and needling manipulation because these are what the acupuncturist readers are interested in. Our assessment based on CONSORT were in accordance with Chen Bo’s study [58], which implied attention should also be paid to methodological issues for the future clinical investigator. However, it should be borne in mind that though most aspects were ‘mentioned’ in trials, usually it was reported very briefly and insufficient information was provided to judge whether or not it was conducted properly. Therefore, the reporting quality was actually lower than it appeared in Table S4 and Table S5. Adequate training of researchers and better dissemination of CONSORT/STRICTA by editors of Chinese journals are also warrant.

In summary, the reported beneficial effect of manual acupuncture for DPN cannot be taken as confirmative conclusion. To ensure evidence-based clinical practice, further rigorous placebo-controlled, randomized trials are warranted. For future trials, more attention should be paid to reducing risks of bias, and the reporting quality should be improved by complying with the CONSORT and STRICTA statements.

## Supporting Information

Figure S1 **Forest plot of comparison of manual acupuncture versus conventional medicine for the outcome of global symptom improvement.**
(DOCX)

Figure S2 **Forest plot of comparison of manual acupuncture plus conventional medicine versus conventional medicine for the outcome of global symptom improvement.**
(DOCX)

Figure S3 **Funnel plot of comparison of manual acupuncture versus conventional medicine for the outcome of global symptom improvement.**
(DOCX)

Table S1 **Characteristics of included RCTs.**
(DOCX)

Table S2 **Detailed information of treatment in included trials.**
(DOCX)

Table S3 **Effect estimations of manual acupuncture for treatment of DPN in included trials.**
(DOCX)

Table S4 **Results of evaluation of included RCTs based on 25 standards of CONSORT 2010.**
(DOC)

Table S5 **Results of evaluation of included RCTs based on 6 standards of STRICTA 2010.**
(DOCX)

Checklist S1 **PRISMA 2009 checklist.**
(DOC)
