# Correction: Manual Acupuncture for Treatment of Diabetic Peripheral Neuropathy: A Systematic Review of Randomized Controlled Trials

**DOI:** 10.1371/journal.pone.0091110

**Published:** 2014-03-10

**Authors:** The PLOS ONE Staff

Information regarding funding for the fourth author is incorrectly omitted from the Funding statement. The Funding statement should read:

This work was supported by the Program for Innovative Research Team of Beijing University of Chinese Medicine (2011-CXTD-09) and the 111 Project (No. B08006). Jian-Ping Liu was partially funded by the grant number 2011ZX09302-006-01-03(5) by the Ministry of Science and Technology of China. Eric Manheimer was partially funded by grant no. R24 AT001293 from the National Center for Complementary and Alternative Medicine (NCCAM) of the US National Institutes of Health. The contents of this article are solely the responsibility of the authors and do not necessarily represent the official views of the NCCAM or the National Institutes of Health. The funders had no role in study design, data collection and analysis, decision to publish, or preparation of the manuscript.
