# Supplementary material for: Manual Acupuncture for Treatment of Diabetic Peripheral Neuropathy: A Systematic Review of Randomized Controlled Trials
Source: PLoS One. 2013 Sep 12;8(9):e73764. doi: 10.1371/journal.pone.0073764 (PMC3771980; doi:10.1371/journal.pone.0073764)
Supplement: Table S1 — Characteristics of included RCTs. (DOCX) [file pone.0073764.s004.docx]

**Table S1.** Characteristics of included RCTs

| **Study ID** | **No. (M/F)** | | **Mean age (yesrs)** | | **Intervention** | **Outcomes** |
| --- | --- | --- | --- | --- | --- | --- |
|  | **T** | **C** | **T** | **C** |  |  |
| Chen 2011 | 20(12/8) | 20(11/9) | 52(38-65) | 5.07( 41-61) | T: Manual acupuncture;  C: Mecobalamin | Global symptom improvement;  MNCV and SNVC in common peroneal and median nerve; |
| Deng 2011 | 42(26/16) | 44(22/22) | 45.4(35-70) | 45.4 (35-70) | T: Manual acupuncture  C: No treatment | Global symptom improvement;  MNCV and SNVC in common peroneal and median nerve; SNVC in ulnar nerve |
| Fei 2011 | 30(17/13) | 30(19/11) | 54.3+0.6 | 55.4+0.7 | T: Manual acupuncture;  C: Mecobalamin | Global symptom improvement;  MNCV and SNVC in tibial nerve |
| Ji 2010 | 40(22/18) | 40(23/17) | 60.78+4.26 | 62.24+4.13 | T: Manual acupuncture;  C: Mecobalamin | Global symptom improvement; |
| Li 1998 | 31(17/14) | 22(12/10) | NA | NA | T: Manual acupuncture  C: Vitamin B1 and B12 | Global symptom improvement; |
| Li 2005 | 30(17/13) | 30(20/10) | 56.1+3.2 | 56.1+3.2 | T: Manual acupuncture plus mecobalamin;  C: Mecobalamin | Global symptom improvement;  MNCV in common peroneal and median nerve; |
| Li 2011 | 50(28/22) | 50(24/26) | 58.92+5.24 | 58.92+5.24 | T: Manual acupuncture;  C: Mecobalamin | Global symptom improvement;  MNCV in common peroneal and ulnar nerve; |
| Li 2011a | 14 | 14 | NA | NA | T: Manual acupuncture plus mecobalamin;  C: Mecobalamin | Global symptom improvement; |
| Luo 2010 | 30(16/14) | 30(13/17) | 61.97+4.75 | 61.60+4.11 | T: Manual acupuncture;  C: Mecobalamin | Global symptom improvement;  MNCV and SNVC in left and right tibial nerve |
| Ma 2010 | 34(20/14) | 34(16/18) | 45.4+7.6 | 45.4+7.6 | T: Manual acupuncture;  C: Mecobalamin | Global symptom improvement; |
| Qiang 2009 | 40(22/19) | 40(23/18) | 63.20+8.72 | 63.08+8.61 | T: Manual acupuncture;  C: Mecobalamin | Global symptom improvement; |
| Ren 2007 | 30(18/12) | 30(21/9) | 63.3+12.58 | 60.8+11.47 | T: Manual acupuncture  C: Vitamin B1 and B12 | Global symptom improvement;  MNCV in common peroneal and median nerve; |
| Song 2005 | 22(14/8) | 20(13/7) | 58.92+5.24 | 58.92+5.24 | T: Manual acupuncture  C: Vitamin B1, B6 and B12 | Global symptom improvement;  MNCV in common peroneal and ulnar nerve; |
| Wang 2001 | 19(10/11) | 17(11/4) | 56.12(49-76) | 56.12(49-76) | T: Manual acupuncture  C: Vitamin B1 and B12 | Global symptom improvement; |
| Wang 2006 | 36(18/18) | 34(18/16) | 58.5(35-82) | 59(37-80) | T: Manual acupuncture  C: Vitamin B12 | Global symptom improvement;  MNCV and SNVC in right common peroneal and left median nerve |
| Wang 2007 | 50(30/20) | 30(20/10) | 55.8(29-73) | 56.1(27-74) | T: Manual acupuncture  C: No treatment | MNCV and SNVC in median and tibial nerve |
| Wang 2010 | 34(20/14) | 32(18/14) | 56.10+5.33 | 58.45+8.52 | T: Manual acupuncture plus mecobalamin;  C: Mecobalamin | MNCV and SNVC in common peroneal and median nerve |
| Xu 2003 | 34(15/19) | 28(11/17) | 53.62+11.17 | 54.98+12.03 | T1: Manual acupuncture  T2: Tapping collaterals with skin needles  C: No treatment | Global symptom improvement; |
| Yan 2007 | 46(24/22) | 42(22/20) | 46.7-74.98 | 46.5-75.6 | T: Manual acupuncture plus mecobalamin;  C: Mecobalamin | Global symptom improvement;  MNCV and SNVC in common peroneal nerve |
| Yuan 2008 | 40(18/22) | 39(19/20) | 35-71 | 36-69 | T: Manual acupuncture  C: No treatment | Global symptom improvement; |
| Zhang 2007 | 32(13/19) | 33(15/18) | 52.0 | 53.0 | T: Manual acupuncture  C: Inositol | Global symptom improvement; |
| Zhao 2001 | 23(11/13) | 23(10/12) | 52.41+4.06 | 51.76+4.14 | T: Manual acupuncture;  C: Mecobalamin | Global symptom improvement;  MNCV and SNVC in common peroneal, median and tibial nerve |
| Zhao 2007 | 30(16/14) | 30(16/14) | 62.30+7.33 | 62.17+7.93 | T: Manual acupuncture;  C: Mecobalamin | Global symptom improvement;  MNCV and SNVC in tibial nerve |
| Zhao 2008 | 20(8/12) | 20(12/8) | 54.5+11.3 | 57.8+11.9 | T1: Manual acupuncture;  T2: Acupuncture and moxibustion  C: Mecobalamin | Global symptom improvement; |
| Yao 2012 | 40(24/16) | 40(21/19) | 54.5(38-77) | 53.4(37-72) | T: Manual acupuncture;  C: Mecobalamin | Global symptom improvement;  MNCV and SNVC in common peroneal nerve |

**Abbreviations:** T, Intervention group; C, control group; MNCV, motor nerve conduction velocity; SNCV, sensory nerve conduction velocity.
