# Supplementary material for: Manual Acupuncture for Treatment of Diabetic Peripheral Neuropathy: A Systematic Review of Randomized Controlled Trials
Source: PLoS One. 2013 Sep 12;8(9):e73764. doi: 10.1371/journal.pone.0073764 (PMC3771980; doi:10.1371/journal.pone.0073764)
Supplement: Table S2 — Detailed information of treatment in included trials. (DOCX) [file pone.0073764.s005.docx]

**Table S2.** Detailed information of treatment in included trials.

| **Study ID** | **Intervention** | **Control** |
| --- | --- | --- |
| Chen 2011 | The main acupoints included Yishu (MBW12), Zusanli (ST36), Huantiao (GB30), Yanglingquan (GB34) , and according to TCM syndromes added the assisted acupoints including 1-2 from Ashi point, Quchi (LI11), Sanyinjiao (SP6), Taixi (KI3). After obtaining qi (de qi), manipulated the needles in Pishu (UB20), Zusanli (ST36) with twirling reinforcing method (ti cha zhuan bu fa), and in Huantiao (GB30), Yanglingquan (GB34) with twirling reducing method (ti cha nian zhuan xie fa), for one minute. Retained the needles for 20 minutes, and manipulated them one time before taking out the needles. For cold deficiency syndrome, moxibustion could be used, and blood-letting was employed when patients felt heat. 4 weeks in total. | Mecobalamin tablet, orally, three times per day, 500μg/day, 4 weeks in total. |
| Deng 2011 | The acupoints included Hegu (LI4), Waiguan (SJ5), Quchi (LI11) and Jianyu (LI15) of the upper limbs, and Taixi (KI 3), Jiexi (ST 41), Zusanli (ST 36), Yanglingquan (GB34), Sanyinjiao (SP 6), and Neiting (ST 44) of the lower limbs. Applied No. 28 filiform needle (1.5-2.5 *cun*in length). Retained needles for 40 min, once par day, ten days as one course, with a two/three-break between courses, 3 courses in total. | No treatment |
| Fei 2011 | The acupoints included Pishu (BL20), Shenshu (BL23), Yishu, Zusanli (ST36) and Sanyinjiao (SP6). Using No. 30 filiform needle (1-1.5 cun in length), and manipulated the needles with neutral supplementation and draining method (ping bu ping xie fa), and with lifting-thrusting and twirling method (ti cha nian zhuan) for obtaining qi (de qi). Retained the needles for 30 minutes, once per day, ten times as one course, three courses in total. | Mecobalamin tablet, orally, three times per day, 500μg/day, 30 days in total. |
| Ji 2010 | The acupoints included Quchi (LI 11), Hegu (LI 4), Zhongwan (RN 12), Xuehai (SP10), Diji (SP 8), Zusanli (ST36), Yinlingquan (SP 9), Yanglingquan (GB 34), Fenglong (ST40), Sanyinjiao (SP6) and Taichong (LV3). Manipulated the needles with neutral supplementation and draining method (ping bu ping xie fa), and retained the needles for 30min, once per day, 4 weeks in total. | Mecobalamin injection, intramuscular injection, once per day, 500μg/day, 4 weeks in total. |
| Li 2011 | The acupoints included Shenshu (BL23), Feishu (BL13), Zusanli (ST36), Sanyinjiao (SP6); for numbness in lower limbs, added Xuanzhong (GB39), Weizhong (BL54), Yanglingquan (GB34); for numbness in upper limbs, added Quchi (LI11), Shousanli (LI10). Manipulated needles with "Compound Shaoshan Manipulation". After obtaining qi (de qi), retained needles for 20 to 30 minutes, once per day, 1 month in total. | Mecobalamin injection, intramuscular injection, three times per week, 500μg, 1 month in total. |
| Li 1998 | The acupoints included Zusanli (ST36), Sanyinjiao (SP6), Yanglingquan (GB34), Fenglong (ST40), Quchi (LI11), Taibai (SP3) on both sides. Maniputated needles with Even reinforcing and reducing manipulation (ping bu ping xie). After developing needle sensation, retained the needles for 20 min, once per day, with a two-day break after every continuous 10 times of treatment, 30 times in total. | Vitmin Bi and B12 injection, intramuscular injection, (usage and dosage not reported), 30 days in total. |
| Li 2005 | 1. Acupuncture: The acupoints included Dazhui (DU14), Pishu (BL20), Shenshu (BL23), Quchi (LI11), Zusanli (ST36), Neiguan (PC6), Hegu (LI4), Yanglingquan (GB34), Xuanzhong (GB39) and Sanyinjiao (SP6) as main acupoints, and selected Ashi point as assisted acupoints from the pain location. Manipulated the needles with neutral supplementation and draining method (ping bu ping xie fa), and lifting-thrusting and twirling method (ti cha nian zhuan) for obtaining qi (de qi) and to promote needle sensation arriving to extremities and the needles should be retained for 20 to 30 minutes, once a day, 2 months as a course, and for 2 courses in total. If there were some complications, treated symptomatically. 2.Mecobalamine injection, intramuscularly, once per day, 100mg/day, 8 weeks in total. | Mecobalamine injection, intramuscular injection, once per day, 100mg/day, 8 weeks in total. |
| Li 2011a | 1. Acupuncture: The acupoints included Hegu (LI 4), Houxi (SI 3), and Quchi (LI 11) of the upper limbs, Yongquan (KI 1), Jiexi (ST 41), Sanyinjiao SP 6), Diji (SP 8), Yinlingquan (SP 9), Zusanli (ST 36), Yanglingquan (GB 34) of the lower limbs, and Xiawan (RN 10), Zhongwan (RN 12), Qihai (RN 6), Guanyuan (RN 4), Ganshu (BL 18), Pishu (BL 20) and Shenshu (BL 23) of the back. After obtaining qi (de qi), manipulated the needles with neutral supplementation and draining method (ping bu ping xie). Retained the needle for 15 min, once per day, 45 days in total. 2. Mecobalamin tablet, 500μg/tablet, three times per day, 45 days in total. | Mecobalamin tablet, orally, three times per day, 500μg/day, 45 days in total. |
| Luo 2010 | 1. Acupuncture: The main points included Pishu (BL20), Shenshu (BL23), Yishu, Quchi (LI11), Zusanli (ST36), Geshu (BL17) and Xuehai (SP10). For syndrome of deficiency of both qi and yin added Sanyinjiao (SP6) and Taixi (KI3); for deficiency of both yin and yang syndrome, added Qihai (RN6) and Guanyuan (RN4); for the numbness and pain in the upper extremities, added Hegu (LI4), Neiguan (PC6) and Baxie (EX-UE9) of two sides; for in the numbness and pain lower extremities added Rangu (KI2), Taichong (LV3) and Bafeng (EX-LE10); local area: Ashi points. Manipulated needles at Pishu (BL20), Shenshu (BL23) and Yishu with lifting-thrusting and twirling method (ti cha nian zhuan), and other points with neutral supplementation and draining method (ping bu ping xie fa). After obtaining qi (de qi), retained needles for 30 min. Two times of manipulation, once per day, 10 times as one course, with a two-day break between courses, 3 courses in total. | Mecobalamin injection, intramuscular injection, once per day, 500μg/day, 30 days in total. |
| Ma 2010 | The acupoints included Quchi (LI11), Hegu (LI4), Xuehai (SP10), Qihai (BL24), Neiguan (P6), Geshu (BL17),Zusanli (ST-36), Xuanzhong (GB30), Sanyingjiao (SP6) et al. Using No. 28 filliform needles (1-1.5 *cun* in length), maniputed needles with Even Reinforming and reducing Methods (ping bu ping xie), and lifting-thrusting and twirling method, once every other 10 minutes. After obtaing qi (de qi), retained the needles for 30 mintues. Two weeks as one course, with a three-day break between courses, 3 courses in total. | Mecobalamin Tablets, orally, three times per day, 500μg per time, 6 weeks in total. |
| Qiang 2009 | The acupoints included Ganshu (BL18), Pishu (UB20), Geshu (BL17), Quchi (LI11), Shousanli (LI10), Waiguan (TW5), Hegu (LI4), Xuehai (SP10), Yanglingquan (GB34), Zusanli (ST-36), Sanyinjiao (SP6), Taichong (LV3), and Taixi (LI3). Applied Huatuo acupuncture needles (25mm in length, diameter 0.35mm), and after obtaining qi (de qi), retained the needles for 30 minutes, and twirled needles once every 15 minutes, 6 treatments per week, 4 weeks in total. | Mecobalamin injection, intramuscular injection, once per day, 500μg per time, 4 weeks in total. |
| Ren 2007 | The acupoints included Fengchi (GB20), Quchi (LI11), Waiguan (TW5), Zusanli (ST-36), Weizhong (BL54), Yanglingquan (GB34), Fenglong (ST40), Sanyinjiao (SP6), Taichong (LV3) and Xuehai (SP10); for symptoms in lower limbs, added Bafeng (EX-LE10), Jiexi (ST41), Xuanzhong (GB39) and Chengshan (BL57); for symptoms in upper limbs, added Baxie (EX-UE9), Hegu (LI4), Shousanli (LI10). Manipulated slowly with moderate stimulation by neutral supplementation and draining method (ping bu ping xie). If patients had strong needle sensation, retained the needles for 20 minutes. Once per day, 10 times as one course, with a two-day break between courses, 30 times in total. | Vitamin B1 and B12 injection, intramuscular injection, once per day, Vitamin B1 100mg and B12 500μg per time, 30 days in total. |
| Song 2005 | The main acupoints included Pishu (BL20), Feishu (BL13), Zusanli (ST36) and Sanyinjiao (SP6) of both sides. For numbness and pain in lower extremities, added Chengshan (BL57), Chengjin (BL56), Weizhong (BL40), Yanglingquan (GB34) of both sides; for in upper extremities, added Quchi (LI11), Shousanli (LI10) of both sides. Manipulated the needles with neutral supplementation and draining method (ping bu ping xie fa), and after obtaining qi (de qi) retained needles for 20 to 30 min, once per day, 1 month in total. | Vitamin B12 injections, intramuscular injection, 500μg per time, 3 times per week; Vitamin B1 and Vitamin B6 tablets, orally, 20mg respectively, three times per day, 1 month in total. |
| Wang 2001 | The main acupoints included Guanyuan (RN4), Sanyinjiao (SP6)(both sides), Yinlingquan (SP9)(both sides), Xuehai (SP10)(both sides), Zusanli (ST36)(both sides), Taixi (KI3)(both sides), Taichong (LV3)(both sides). For the upper extremities, added Jianyu (LI15), Quchi (LI11) and Waiguan (SJ5); for the lower extremities, added Huantiao (GB30), Yanglingquan (GB34) and Kunlun (BL60). Manipulated the needles with neutral supplementation and draining method (ping bu ping xie fa), and after obtaining qi (de qi) retained needles for 30 to 40 minutes, once per day, six times per week with one-day break between weeks, 8 weeks in total. | Vitamin B1 (100mg) and Vitamin B12 (500μg) injection, intramuscular injection, once per day, 8 weeks in total. |
| Wang 2006 | For numbness and pain in upper extremities, selected jing-well point, Shaoshang (LU11), Shangyang (LI1), Shaochong (HT9), Zhongchong (PC9), Shaoze (SI1) and Guanchong (SJ1) as main points and Er’jian (LI2), Sanjian (LI3), Yangxi (LI5), Quchi (LI11), Yemen (SJ2), Zhongzhu (SJ3), Qiangu (SI2), Houxi (SI3), Yuji (LU10) and Daling (PC7) as assisted points; for numbness and pain in the lower extremities, selected jing-well point, Yongquan (KI1), Lidui (ST45), Yinbai (SP1), Zuqiaoyin (GB44), Dadun (LV1) and Zhiyin (BL67) as main points and Rangu (KI2), Taixi (KI3), Yingu (KI10), Xingjian (LV2), Taichong (LV3), Zhongfeng (LV4), Ququan (LV8), Neiting (ST44) and Jiexi (ST41) as assisted points. If lesions were in the back of hands and feet, the five transport points on the three yang channels were selected as main points. If lesions were in the palms or the soles of feet, the five transport points on the three yin channels were selected as main points. Applied a three-edged needle at the jing-well point for blood-letting about 4 to 6 drops; then needled at the ying-spring point, shu-stream points, jing-river point and he-sea point. Manipulated Yishu, Pishu (BL20) and Zusanli (ST36) with neutral supplementation and draining method (ping bu ping xie fa). After obtaining qi (de qi) retained needles for 30 minutes, once every two days, ten times as one course, with a 10-day break between courses, 30 times in total. | Vitamin B12 (0.5mg) injection, intramuscular injection, once per day, 30 days in total. |
| Wang 2007 | For the upper extremities, selected Hegu (LI4), Neiguan (PC6), Yangchi (SJ4) and Quchi (LI11); for the lower extremities, selected Taichong (LV3), Jiexi (ST41), Zusanli (ST36) and Yanglingquan (GB34), once per day for 40 minutes, manipulated once per time. 30 days in total. | No treatment |
| Wang 2010 | 1. Acupuncture: The acupoints included Zusanli (ST-36), Jiexi (ST41), Neiting (ST44), Quchi (LI11), Taixi (KI3), Guanyuan (CV4),Yanglingquan (GB34), Geshu (BL17), Sanyinjiao (SP6), Ashi point. Applied No.28 filliform needles (1.5-2.5 *cun* in length). After obtaining qi (de qi), retained the needles for 30 minutes, and manipulated with moderate stimulation once every 5 minutes by neutral supplementation and draining method (ping bu ping xie fa), once per day, 4 weeks in total. 2. Mecobalamin injection, intramuscularly, once per day, 500μg per time, 4 weeks in total. | Mecobalamin injection, intramuscular injection, once per day, 500μg per time, 4 weeks in total. |
| Xu 2003 | The acupoints included Yishu, Zusanli (ST36) and Quchi (LI11) (both sides) with supplementation method; for the lower extremities added Bafeng (EX-LE10) with the draining method; for the upper extremities added Baxie (EX-UE9) with the draining method. After obtaining qi (de qi), retained needles for 30 minutes and manipulated them every 10 minutes with twirling method, once every two days, 10 times as one course, 20 times in total. | No treatment |
| Yan 2007 | 1. Acupuncture: for lesions in lower extremities, selected Taichong (LV3), Neiting (ST44), Jiexi (ST41), Xingjian (LV2), Sanyinjiao (SP6), Yinlingquan (SP9) and Yanglingquan (GB34); for lesions in upperr extremities, selected Erjian (LI2), Sanjian (LI3), Yangxi (LI5), Quchi (LI11), Yemen (SJ2), Zhongzhi (SJ3), Qiangu (SI2), Houxi (SI3) and Daling (PC7); for lesions in the back of hands and feet, the points on the three yang channels were selected as main points; for lesions in the palms or the soles of feet, the points on the three yin channels were selected as main points. Selected 4 to 5 points per time, and Yishu, Pishu (BL20) and Zusanli (ST36) with lifting-thrusting and twirling method (ti cha nian zhuan fa). Manipulated the needles with neutral supplementation and draining method (ping bu ping xie fa), and after obtaining qi (de qi) retained needles for 20 minutes, once a day,15 days as one course, with a three-day break between courses. 2. Mecobalamin tablet, orally, three times per day, 500μg/day, 3 months in total. | Mecobalamin tablet, orally, three times per day, 500μg/day, 3 months in total. |
| Yuan 2008 | The main acupoints included Zusanli (ST36), Sanyinjiao (SP6), Pishu (BL20), Weishu (BL21), Yishu (MBW12), Feishu (BL13), Ganshu (BL18), Shenshu (BL23), Fenglong (ST40), Yanglingquan (GB34), Guanyuan (CV4) and Qihai (BL24). For the upper extremities, added Jianyu (LI15), Jianzhen(SI9), Binao(LI14), Quchi (LI11), Shousanli (LI10) and Geshu (BL17); for the upper extremities, added Biguan (ST 31), Futu (ST32), Liangqiu (ST34), Neiting(ST44) and Xiangu(ST43). For severe numbness of hands and feet, added Bafeng(EX-LE10) and Baxie(EX-UE9), or using plum-blossom needle to tap the local areas; for blood stasis, added Xuehai (SP10) and Geshu (BL17). Applied No. 30 filiform needle (1 to 1.5 *cun* in length). After obtaining qi (de qi), retained the needles for 30 minutes, and manipulated the needles with needling direction for supplementation/drainage (ying sui bu xie fa), once every 10 minutes, once per day, 10 times as one course for five courses with a two-day break, 50 times in total. | No treatment |
| Zhang 2007 | The main points included Ganshu (BL18), Pishu (BL20), Shenshu (BL23),Yishu (MBW12), Feishu (BL13), Zusanli (ST 36), Sanyinjiao (SP6), Taibai (SP3), Zutonggu(BL66), Qihai (BL24), Guanyuan (CV4), Fenglong (ST40) and Yanglingquan (GB34). Added points according to syndromes: Jianyu (LI 15), Quchi (LI11), Shousanli (LI10), Geshu (BL17), Biguan(ST 31), Futu (ST32), Liangqiu (ST34), Neiting(ST44) and Xiangu (ST43). For blood stasis, added Xuehai(SP10) and Geshu (BL17); for turbid phlegm, added Yinlingquan (SP9) and Diji (SP 8); for severe numbness of hands and feet, added Bafeng(EX-LE10) and Baxie (EX-UE9). Applied No. 30 filiform needle (1 to 1.5 *cun* in length). Manipulated the needles with neutral supplementation and drainage method (ping bu ping xie fa); used even lifting-thrusting and twirling method for obtaining qi (de qi), and btaining qi (de qi) retained needles for 25 min, once par day, 14 times as one course for five courses with a four-day break between courses, 60 times in total. | Inositol tablet, orally, three times per day, 2g/day, 3 months in total. |
| Zhao 2001 | The acupoints included Shenshu (BL23), Guanyuan (RN4), Qihai (RN6), the 7 to 12 Jiaji (EX-B2), Ganshu (BL18), Geshu (BL17), Sanyinjiao (SP6), Taixi (KI3) and Zhaohai (KI6) as main points. For severe symptoms in the upper extremities, added Quchi (LI11), Waiguan (SJ5) and Hegu (LI4); for the lower extremities, added Zusanli (ST36), Xuehai (SP10), Yinlingquan (SP9) and Bafeng (EX-LE10). Manipulated the needles with neutral supplementation and draining method (ping bu ping xie fa), and after obtaining qi (de qi) retained needles for 20 minutes, during which manipulated the needle two times wih interval. Once per day, 10 times as one course for five courses with a three/five-day break between courses, 50 times in total. | Mecobalamin tablet, orally, three times per day, 500μg/day, 8 weeks in total. |
| Zhao 2007 | The acupoints included Ganshu (BL18), Feishu (BL13), Pishu (UB20), Yishu (MBW12), Shenshu (BL23), Taixi (KI3), Quchi (LI11), Hegu (LI4), Zusanli (ST 36), Sanyinjiao (SP6) and Yanglingquan (GB34). For deficiency of both qi and yin, added Guanyuan (CV4) and Qihai (BL24); for deficiency of both yin and yang, added Guanyuan (CV4), Qihai (BL24) and Mingmen (GV4); for blood stasis, added Geshu (bl17) and Qihai (BL24). Manipulated the needles with neutral supplementation and drainage (ping bu ping xie fa), and manipulated twice intermittently. After obtaining qi (de qi), retained the needles for 30 minutes. Once every other day, 10 times as one course for 3 courses, 2 months in total. | Mecobalamin tablet, orally, three times per day, 500μg/day, 2 months in total. |
| Zhao 2008 | The main points included Shenshu (BL23), Pishu (BL20), Zusanli (ST36). Assisted points: for numbness and pain in the upper extremities, added Quchi (LI11) and Waiguan (SJ 5); for in the lower extremities, added Sanyinjiao (SP6) and Taixi (KI3). After inserting needles (0.30 mm*40 mm), applied lifting-thrusting and twriling method. Used supplementation method on Shenshu (BL23) and Pishu (UB20), and drainage method for other points. After obtaining qi (de qi), retained the needles for 30 minutes. Once every other day, 10 times as one course for 2 coueses with a two-day break between courses, 40 days in total. | Mecobalamin tablet, orally, three times per day, 500μg/day, 40 days in total. |
| Yao 2012 | The points included Neiguan (PC6), Gongsun (SP 4), Waiguan (SJ5), Zulinqi (GB 41), Houxi (SI 3), Shenmai (BL 62), Lieque (LU 7) and Zhaohai (KI6). Using No. 28 filiform needles inserted into points with 0.5-1 cun. Manipulated the needles with neutral supplementation and draining method (ping bu ping xie fa), and after obtaining qi (de qi) retained needles for 30 minutes, during which manipulated the needle two times wih interval. Once per day, 4 weeks as one course for 2 courses. | Mecobalamin tablet, orally, once per day, 500μg/day, 8 weeks in total. |
