# Supplementary material for: Manual Acupuncture for Treatment of Diabetic Peripheral Neuropathy: A Systematic Review of Randomized Controlled Trials
Source: PLoS One. 2013 Sep 12;8(9):e73764. doi: 10.1371/journal.pone.0073764 (PMC3771980; doi:10.1371/journal.pone.0073764)
Supplement: Table S3 — Effect estimations of manual acupuncture for treatment of DPN in included trials. (DOCX) [file pone.0073764.s006.docx]

**Table S3.** Effect estimations of manual acupuncture for treatment of DPN in included trials

| **Outcomes** | **Effect estimate (95%CI)** | **Study ID** |
| --- | --- | --- |
| ***Manual acupuncture vs. mecobalamin*** | | |
| Global symptom improvement | RR 1.31 (1.21, 1.42) | Chen 2011, Fei 2011, Ji 2010, Li 2011, Luo 2010, Ma 2010, Qiang 2009, Zhao 2001, Zhao 2007, Zhao 2008, Yao 2012 |
| Change of MNCV in common peroneal nerve | MD 6.25 (5.53, 6.97) | Chen 2011, Li 2011, Zhao 2001, Yao 2012 |
| Change of SNCV in common peroneal nerve | MD 3.53 (2.66, 4.40) | Chen 2011, Zhao 2001, Yao 2012 |
| Change of MNCV in median nerve | MD 3.30 (2.10, 4.49) | Chen 2011, Zhao 2001 |
| Change of SNCV in median nerve | MD 1.82 (0.82, 2.83) | Chen 2011, Zhao 2001 |
| Change of MNCV in tibital nerve | MD 2.02 (1.18, 2.86) | Fei 2011, Zhao 2001, Zhao 2007 |
| Change of SNCV in tibital nerve | MD 2.31 (1.59, 3.02) | Fei 2011, Zhao 2001, Zhao 2007 |
| Change of MNCV in left tibital nerve | MD 1.74 (1.44, 2.04) | Luo 2010 |
| Change of SNCV in left tibital nerve | MD 1.54 (1.17, 1.91) | Luo 2010 |
| Change of MNCV in right tibital nerve | MD 1.48 (1.16, 1.80) | Luo 2010 |
| Change of SNCV in right tibital nerve | MD 1.53 (1.19, 1.87) | Luo 2010 |
| Change of MNCV in ulnar nerve | MD 11.32 (10.09, 12.55) | Li 2011 |
| ***Manual acupuncture vs. Vitamin B1 and B12*** | | |
| Global symptom improvement | RR 1.55 (1.33, 1.80) | Li 1998, Ren 2007, Song 2005, Wang 2001, Wang 2006 |
| Change of MNCV in common peroneal nerve | MD 7.56 (5.98, 9.13) | Ren 2007, Song 2005 |
| Change of MNCV in right common peroneal nerve | MD 8.17 (5.46, 10.88) | Wang 2006 |
| Change of SNCV in right common peroneal nerve | MD 8.50 (5.82, 11.18) | Wang 2006 |
| Change of MNCV in median nerve | MD 3.72 (0.98, 6.46) | Ren 2007 |
| Change of MNCV in left median nerve | MD 12.50 (9.50, 15.50) | Wang 2006 |
| Change of SNCV in left median nerve | MD 14.52 (11.97, 17.07) | Wang 2006 |
| Change of MNCV in ulnar nerve | MD 11.32 (9.44, 13.20) | Song 2005 |
| ***Manual acupuncture vs. inositol*** | | |
| Global symptom improvement | RR 1.38 (1.03, 1.84) | Zhang 2007 |
| ***Manual acupuncture vs. no treatment*** | | |
| Global symptom improvement | RR 1.56 (1.31, 1.85) | Deng 2011, Xu 2003, Yuan 2008 |
| Change of MNCV in common peroneal nerve | MD 4.10 (0.09, 8.11) | Deng 2011 |
| Change of SNCV in common peroneal nerve | MD 7.30 (3.09, 11.51) | Deng 2011 |
| Change of MNCV in median nerve | MD 7.42 (5.32, 9.52) | Deng 2011, Wang 2007 |
| Change of SNCV in median nerve | MD 4.74 (2.92, 6.55) | Deng 2011, Wang 2007 |
| Change of MNCV in tibital nerve | MD 7.80 (5.40, 10.20) | Wang 2007 |
| Change of SNCV in tibital nerve | MD 6.18 (3.15, 9.21) | Wang 2007 |
| Change of SNCV in ulnar nerve | MD 2.10 (-1.30, 5.50) | Deng 2011 |
| ***Manual acupuncture + mecobalamin vs. mecobalamin*** | | |
| Global symptom improvement | RR 1.56 (1.28, 1.90) | Li 2005, Li 2011a, Yan 2007 |
| Change of MNCV in common peroneal nerve | MD 4.93 (3.92, 5.94) | Li 2005, Wang 2010, Yan 2007 |
| Change of SNCV in common peroneal nerve | MD 4.03 (2.81, 5.25) | Wang 2010, Yan 2007 |
| Change of MNCV in median nerve | MD 2.79 (1.31, 4.28) | Li 2005, Wang 2007 |
| Change of SNCV in median nerve | MD 2.70 (1.01, 4.39) | Wang 2010 |

## Abbreviations: CI, confidence interval; RR, risk ratio; MD, mean difference; MNCV, motor nerve conduction velocity; SNCV, sensory nerve conduction velocity;
