# Supplementary material for: Manual Acupuncture for Treatment of Diabetic Peripheral Neuropathy: A Systematic Review of Randomized Controlled Trials
Source: PLoS One. 2013 Sep 12;8(9):e73764. doi: 10.1371/journal.pone.0073764 (PMC3771980; doi:10.1371/journal.pone.0073764)
Supplement: Table S5 — Results of evaluation of included RCTs based on 6 standards of STRICTA 2010. (DOCX) [file pone.0073764.s008.docx]

**Table S5.** Reporting quality of 25 RCTs based on STRICTA 2010

| **Item** | **Detail** | **No. of Reported RCTs (%)** |
| --- | --- | --- |
| **1. Acupuncture rationale** | 1a) Style of acupuncture (e.g. Traditional Chinese Medicine, Japanese, Korean, Western medical, Five Element, ear acupuncture, etc) | 25 (100%) |
|  | 1b) Reasoning for treatment provided, based on historical context, literature sources, and/or consensus methods, with references where appropriate | 0 |
|  | 1c) Extent to which treatment was varied | 0 |
| **2. Details of needling** | 2a) Number of needle insertions per subject per session (mean and range where relevant) | 0 |
|  | 2b) Names (or location if no standard name) of points used (uni/bilateral) | 25 (100%) |
|  | 2c) Depth of insertion, based on a specified unit of measurement, or on a particular tissue level | 1 (4%) |
|  | 2d) Response sought (e.g. *de qi* or muscle twitch response) | 21 (84%) |
|  | 2e) Needle stimulation (e.g. manual, electrical) | 24 (96%) |
|  | 2f) Needle retention time | 25 (100%) |
|  | 2g) Needle type (diameter, length, and manufacturer or material) | 9 (36%) |
| **3. Treatment regimen** | 3a) Number of treatment sessions | 25 (100%) |
|  | 3b) Frequency and duration of treatment sessions | 25 (100%) |
| **4. Other components of treatment** | 4a) Details of other interventions administered to the acupuncture group (e.g. moxibustion, cupping, herbs, exercises, lifestyle advice) | 0 |
|  | 4b) Setting and context of treatment, including instructions to practitioners, and information and explanations to patients | 0 |
| **5. Practitioner background** | 5) Description of participating acupuncturists (qualification or professional affiliation, years in acupuncture practice, other relevant experience) | 0 |
| **6. Control or comparator interventions** | 6a) Rationale for the control or comparator in the context of the research question, with sources that justify this choice | 0 |
|  | 6b) Precise description of the control or comparator. If sham acupuncture or any other type of acupuncture-like control is used, provide details as for Items 1 to 3 above. | 25 (100%) |
