# Supplementary material for: Manual Acupuncture for Treatment of Diabetic Peripheral Neuropathy: A Systematic Review of Randomized Controlled Trials
Source: PLoS One. 2013 Sep 12;8(9):e73764. doi: 10.1371/journal.pone.0073764 (PMC3771980; doi:10.1371/journal.pone.0073764)

Study or Subgroup	Experimental Events	Experimental Total	Control Events	Control Total	Weight	Risk Ratio M-H, Fixed, 95% CI
**1.1.1 Manual acupuncture vs. mecobalamin**						
Chen 2011	18	20	13	20	3.8%	1.38 [0.97, 1.97]
Fei 2011	27	30	18	30	5.3%	1.50 [1.09, 2.06]
Ji 2010	38	40	33	40	9.8%	1.15 [0.98, 1.35]
Li 2011	41	50	31	50	9.2%	1.32 [1.03, 1.70]
Luo 2010	27	30	23	30	6.8%	1.17 [0.93, 1.48]
Ma 2010	26	34	16	34	4.7%	1.63 [1.09, 2.43]
Qiang 2009	37	40	30	40	8.9%	1.23 [1.01, 1.51]
Yao 2012	38	40	33	40	9.8%	1.15 [0.98, 1.35]
Zhao 2001	20	23	14	23	4.1%	1.43 [0.99, 2.06]
Zhao 2007	25	30	16	30	4.7%	1.56 [1.08, 2.26]
Zhao 2008	17	20	13	20	3.8%	1.31 [0.90, 1.89]
**Subtotal (95% CI)**		**357**		**357**	**71.1%**	**1.31 [1.21, 1.42]**
Total events	314		240			
Heterogeneity: Chi² = 9.16, df = 10 (P = 0.52); I² = 0%						
Test for overall effect: Z = 6.53 (P < 0.00001)						
**1.1.2 Manual acupuncture vs. Vitamin B1 and B12**						
Li 1998	30	31	15	22	5.2%	1.42 [1.06, 1.90]
Ren 2007	28	30	19	30	5.6%	1.47 [1.10, 1.97]
Song 2005	20	22	14	20	4.3%	1.30 [0.95, 1.78]
Wang 2001	16	19	6	17	1.9%	2.39 [1.22, 4.67]
Wang 2006	33	36	19	34	5.8%	1.64 [1.20, 2.25]
**Subtotal (95% CI)**		**138**		**123**	**22.8%**	**1.55 [1.33, 1.80]**
Total events	127		73			
Heterogeneity: Chi² = 3.33, df = 4 (P = 0.50); I² = 0%						
Test for overall effect: Z = 5.58 (P < 0.00001)						
**1.1.3 Manual acupuncture vs. inositol**						
Zhang 2007	28	32	21	33	6.1%	1.38 [1.03, 1.84]
**Subtotal (95% CI)**		**32**		**33**	**6.1%**	**1.38 [1.03, 1.84]**
Total events	28		21			
Heterogeneity: Not applicable						
Test for overall effect: Z = 2.16 (P = 0.03)						
**Total (95% CI)**		**527**		**513**	**100.0%**	**1.37 [1.27, 1.46]**
Total events	469		334			
Heterogeneity: Chi² = 17.63, df = 16 (P = 0.35); I² = 9%						
Test for overall effect: Z = 8.80 (P < 0.00001)						
Test for subgroup differences: Chi² = 3.57, df = 2 (P = 0.17), I² = 43.9%						

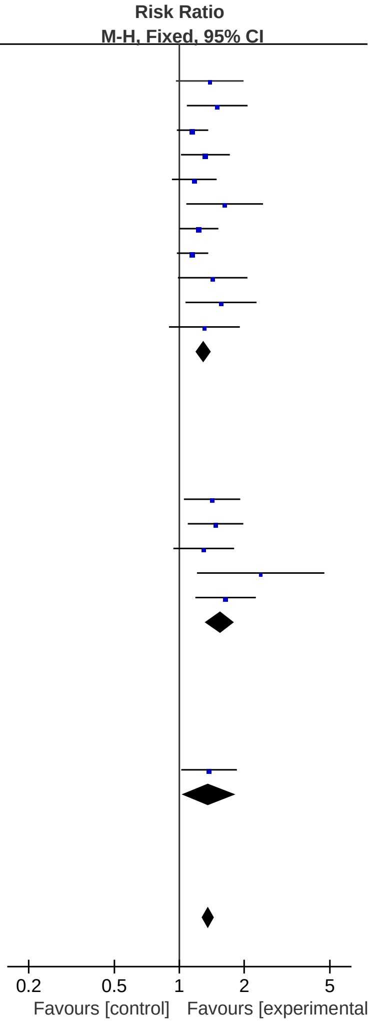

Supplement: Supplementary file 7 [file pone.0073764.s001.docx]
