# Supplementary material for: Manual Acupuncture for Treatment of Diabetic Peripheral Neuropathy: A Systematic Review of Randomized Controlled Trials
Source: PLoS One. 2013 Sep 12;8(9):e73764. doi: 10.1371/journal.pone.0073764 (PMC3771980; doi:10.1371/journal.pone.0073764)

| Study or Subgroup | Experimental Events | Experimental Total | Control Events | Control Total | Weight | Risk Ratio M-H, Fixed, 95% CI |
|---|---|---|---|---|---|---|
| **2.1.1 Manual acupuncture + mecobalamin vs. mecobalamin** | | | | | | |
| Li 2005 | 25 | 30 | 12 | 30 | 24.3% | 2.08 [1.31, 3.32] |
| Li 2011a | 12 | 14 | 5 | 14 | 10.1% | 2.40 [1.15, 5.00] |
| Yan 2007 | 42 | 46 | 31 | 42 | 65.6% | 1.24 [1.01, 1.51] |
| **Subtotal (95% CI)** | | **90** | | **86** | **100.0%** | **1.56 [1.28, 1.90]** |
| Total events | 79 | | 48 | | | |
| Heterogeneity: Chi² = 7.92, df = 2 (P = 0.02); I² = 75% | | | | | | |
| Test for overall effect: Z = 4.39 (P < 0.0001) | | | | | | |
| | | | | | | |
| **Total (95% CI)** | | **90** | | **86** | **100.0%** | **1.56 [1.28, 1.90]** |
| Total events | 79 | | 48 | | | |
| Heterogeneity: Chi² = 7.92, df = 2 (P = 0.02); I² = 75% | | | | | | |
| Test for overall effect: Z = 4.39 (P < 0.0001) | | | | | | |
| Test for subgroup differences: Not applicable | | | | | | |

Risk Ratio
M-H, Fixed, 95% CI

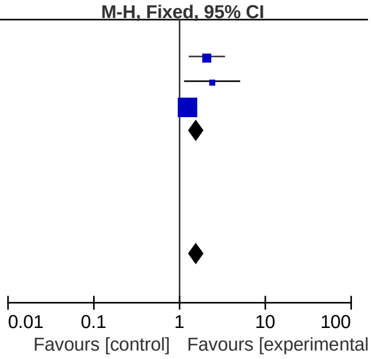

Supplement: Supplementary file 8 [file pone.0073764.s002.docx]
