# Supplementary material for: Manual Acupuncture for Treatment of Diabetic Peripheral Neuropathy: A Systematic Review of Randomized Controlled Trials
Source: PLoS One. 2013 Sep 12;8(9):e73764. doi: 10.1371/journal.pone.0073764 (PMC3771980; doi:10.1371/journal.pone.0073764)
Supplement: Supplementary file 9 [file pone.0073764.s003.docx]

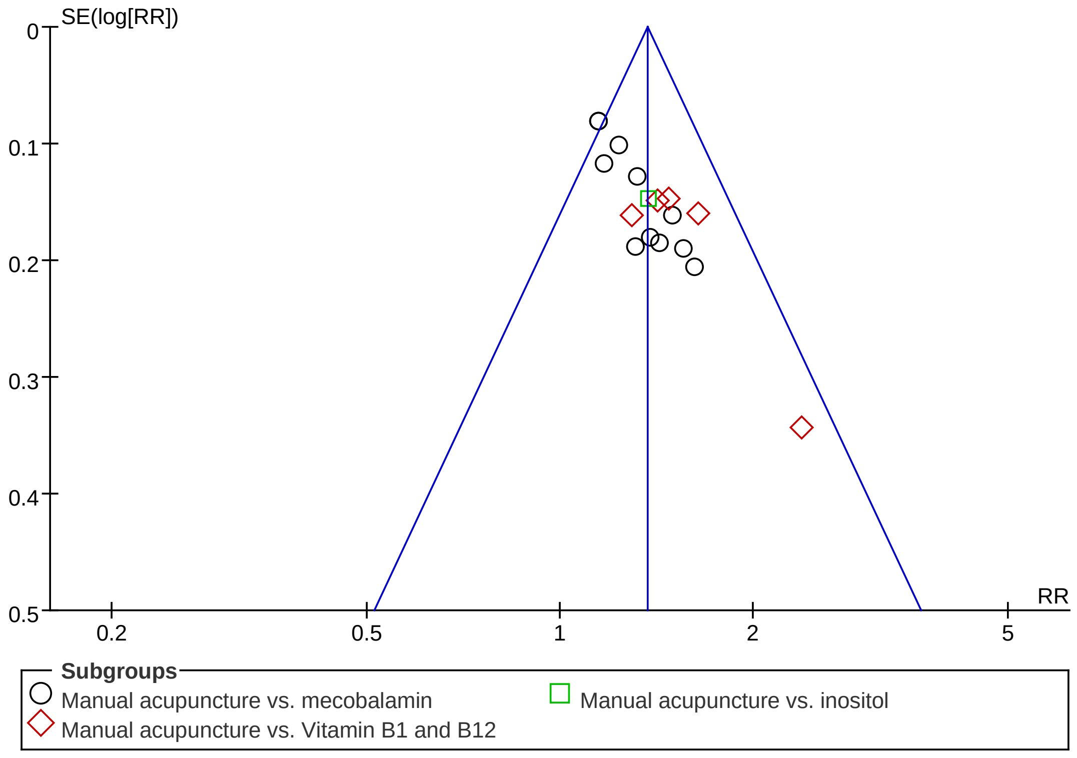
SE(log[RR])
0
0.1
0.2
0.3
0.4
0.5
RR
0.2
0.5
1
2
5
Subgroups
Manual acupuncture vs. mecobalamin
Manual acupuncture vs. inositol
Manual acupuncture vs. Vitamin B1 and B12
